# Self-harm prevalence and associated factors among street children in Mashhad, North East of Iran

**DOI:** 10.1186/s13690-021-00660-x

**Published:** 2021-08-04

**Authors:** Lida Jarahi, Maliheh Dadgarmoghaddam, Atiyeh Naderi, AmirAli Moodi Ghalibaf

**Affiliations:** 1 Community Medicine Department, Faculty of Medicine, Mashhad University of Medical Sciences, Mashhad, Iran; 2 Faculty of Medicine, Mashhad University of Medical Sciences, Mashhad, Iran; 3 Student Research Committee, Faculty of Medicine, Mashhad University of Medical Sciences, Mashhad, Iran

**Keywords:** Self-harm, Street Children, Child Labor, Mental Health Disorder, Mashhad, Iran

## Abstract

**Background:**

Self-harm is intentional harmful behavior in the context of emotional distress. Street children are boys and girls under eighteen who are forced to work or live on the streets. These children are exposed to violent situations and high-risk behaviors like self-harm. This study investigated the prevalence of self-harm in street children in Mashhad, the second Metropolis of Iran.

**Methods:**

In this cross-sectional study, 98 children were assessed with a 22-item of self-harm Inventory (SHI) questionnaire. A trained social worker interviewed the participants who were referred to Mashhad Welfare Office, February-July 2020.

**Results:**

The mean age of participants was 13.8 (2.3) years old, and 71.4 % of them were male. Of street children 59.2 % have had self-harming behavior, among them 8.6 % had one self-harming behavior, and others have more than one. The self-harmed people who had physical injuries, more frequent injuries were hitting (26.5 %), self-starvation (23.5 %), cutting (21.4 %), respectively. In comparison, common psychological injuries were God-distancing (29.6 %) and self-defeating thoughts (19.4 %). The most important risk factors were having a mental disorder (OR = 6.3, *P* = 0.002), losing parents (OR = 4.4, *P* = 0.01), self-harming or suicide history in relatives (OR = 3.2, *P* = 0.001, OR = 4.3, *P* = 0.03 respectively), low-educated parents (OR = 4.2, *P* = 002, OR = 2.8, *P* = 0.02 for father and mother respectively), and age-increasing (OR = 1.5, *P* = 0.001).

**Conclusions:**

The prevalence of self-harming in street children is significantly high. Some of these children are in more high-risk conditions that face them to suffer from self-harming at a younger age. Family factors are more important in predicting self-harming and community health decision-makers should provide educational interventions and psychological support for these children and their families.

## Background

Self-harm is intentional socially unacceptable behaviors that cause harm to the body to overcome the emotional distress, it is described as non-lethal behavior in which a person intentionally injures him/her-self to change him/her current emotions [1]. According to this definition, behaviors such as tattooing, piercing the body, or injuring the skin that is performed according to a particular tradition and culture are not considered a self-harming act [2]. The aim of self-harm in children and teenagers often is not suicide, they just want to reduce inner excitements or attract attention with a simple injury to their skin or body [1]. But self-harm is can become an addictive behavior and it is dangerous and can cause severe physical injury and even death, and also, non-fatal self-harm is a strong predictive factor for suicide attempts [3]. Moreover, self-harm may lead to the person not learning the proper way to deal with stress, feeling guilty, depressed, and ultimately causes exacerbation of the primary psychological illness [4].

The etiology of self-harm refers to many social and psychological factors such as depression, disappointment, low tolerance to stress, low self-esteem, dysfunctional family, community relations, personal problems, psychiatric problems in the family, the impact of peers, rape, physical or psychological abuse, drug abuse, economic poverty and so on [4–6].

Street children are boys and girls under the age of eighteen who are forced to work or live on the streets, especially in large cities to survive. In addition, child labor defined as the work that deprives children of their childhood, and is harmful for their physical and mental development [7, 8]. Too many of these children are working on the streets and are exposed to many high-risk conditions such as abusing, neglecting, antisocial behaviors, prone to physical and mental diseases [7, 8]. Family breakdown, abusing by the family, and running away from home are the most important factors for children to stay on the streets [9]. Due to the conditions experienced by these children, they are more likely to blurt symptoms of depression, anxiety, and stress that are strongly associated with self-harm or suicide [10]. The World Health Organization (WHO) and the United Nations Children’s Fund (UNICEF) estimate the number of child laborers in the world about 250 million between the ages of 5 and 14 that this number is growing [11]. These children potentially exposed to different high-risk situations that cause many problems for their health and for community health. So far, some studies conducted on street child laborers in Iran and other countries, but few studies have expressed self-harming in these children. This study was conducted to determine the prevalence of self-harm and its related factors in Iranian street children in Mashhad, the second Metropolis of Iran.

## Methods

### Study design

We did this cross-sectional study in collaboration with Mashhad Welfare Office to determine the prevalence of self-harm and related factor in street children in Mashhad, Razavi Khorasan province, Iran, in 2020.

### Participants and questionnaire

Participants of this study were street child laborers under the age of 18 years old in Mashhad. All of them were recognized by the Mashhad Welfare Office and referred to investigate their problems and improve their condition.

A trained social worker interviewed street children who were referred to Mashhad Welfare Office and were satisfied to participate in the study. Self-harm was assessed by the self-harm Inventory (SHI) questionnaire, which is a 22-item questionnaire designed in 1998 by Sansone and Wiedermann [12]. This questionnaire evaluates the intentionally done behaviors for a self-harming act, such as drug or alcohol abuse, physical or emotional self-harm, and an abusive relationship. The validity and reliability of the Persian version of the SHI questionnaire were confirmed in previous studies by Tahbaz Hoseinzadeh et al. [13]. Also, at the beginning of the interview, a brief questionnaire about the demographic characteristics of participants, his /her family, and some behaviors that may be risk factors for self-harm was filled.

The Mashhad University of Medical Sciences ethics committee approved the protocol of this survey (no. IR.MUMS.fm.REC.1396.717), and all respondents provided informed consent.

### Sample size and statistical analysis

The study’s sample size was calculated at 98 persons, based on the statistical formula for estimating the frequency of a qualitative variable in the population regarding the prevalence of self-harm in similar studies [1, 3]. Participants were selected by convenience sampling method from all children who were referred to Mashhad Welfare Office between February to July 2020.

Statistical analysis was conducted using SPSS software version 16 (SPSS Inc., Chicago, USA –version 16). Descriptive statistics for the demographic characteristics were presented by the mean and standard deviation for quantitative variables and frequency (percentage) for qualitative variables. Chi-square test and Fisher’s exact test were used to investigate the relationship between qualitative variables. Statistical analysis was conducted using SPSS software version 16 (SPSS Inc., Chicago, USA –version 16). Descriptive statistics for the demographic characteristics were presented by the mean and standard deviation for quantitative variables and frequency (percentage) for qualitative variables. Chi-square test and Fisher’s exact test were used to investigate the relationship between qualitative variables. To compare the mean of quantitative variables in the two groups (children with self-harm and without that), independent t-test or Mann-Whitney tests were used. Logistic regression was used for determining predicting factors of self-harm. A *P*-value less than 0.05 was considered as the significance level.

## Results

Among the Ninety-eight participants, 71.4 % (*n* = 70) were male, and the rest of them (*n* = 28) were female. The age range of participants was 8 to 18 years old, with mean age (Standard deviation) of 13.8 (2.3), and there was no significant difference between male and female in terms of age (*P* = 0.28).

Table 1 shows the characteristics of the participants and their families. According to Table 1 and 25.5 % of street children had dropped out of school, 16.3 % had a history of drug abuse but were now rehabilitated, and 8.2 % were addicted. Based on the past medical history and interviewing, 28.6 % of the participants suffered from mental disorders (anxiety, depression). Among the participants, 23.5 % lost one or both of their parents because of death, and the parents of 8.2 % of children were divorced.

**Table 1:** Individual and family characteristics of the participants by gender comparison

Characteristic	Sub-characteristic	Gender		Total	*P*-Value
		**Male**	**Female**		
		**Number (%)**	**Number (%)**	**Number (%)**	
**Educational grade**	Illiterate	1(1.4)	0(0)	1(1)	0.86
	First elementary	7(10)	3(10.7)	10(10.2)	
	Second elementary	28(40)	14(50)	42(42.9)	
	First high school	20(28.6)	6(21.4)	26(26.5)	
	Second high school	14(20)	5(17.9)	19(19.4)	
**Educational status**	Dropped out of school	17(24.3)	8(28.6)	25(25.5)	0.42
**History of drug abuse**	Addicted	8(11.4)	0(0)	8(8.2)	0.08
	drug rehabilitated	13(18.6)	3(10.7)	16(16.3)	
	Never	49(70)	25(89.3)	74(75.5)	
**History of mental disorders**	Have	21(30.0)	7(25.0)	28(28.6)	0.62
**History of suicide in relatives**	Have	13(18.6)	5(17.9)	18(18.4)	0.59
**History of self-harm in the relatives**	Have	21(30.0)	6(21.4)		0.39
**Parental life status**	Both alive	56(80)	19(67.9)	76(76.5)	0.34
	Only the mother is alive	9(12.9)	7(25)	16(16.3)	
	Only the father is alive	3(4.3)	2(7.1)	5(5.2)	
	Both died	2(2.29)	0(0)	2(2)	
**Parental divorce**	Yes	4(5.7)	4(14.3)	8(8.2)	0.16
**Paternal education Level**	Illiterate	32(45.7)	11(39.3)	43(43.9)	0.83
	Undergraduate	33(47.1)	15(53.6)	48(49)	
	Diploma	5(7.1)	2(7.1)	7(7.1)	
**Maternal education level**	Illiterate	28(40)	10(35.7)	38(38.8)	0.16
	Undergraduate	35(50)	18(64.3)	53(54.1)	
	Diploma	7(10)	0(0)	7(7.1)	

Some of the children did not live with their family and had related with their family rarely (11.2 %). Among the children who related to their parents, about 66 % expressed that they had dysfunctional emotional relationships. Participants reported a history of self-harm in their friends (37 %), siblings (28.6), parents (25.9), and the rest in others. Results showed that 58 people (59.2 %) of the participants reported self-harming behavior.

Table 2 shows the comparison of characteristics’ participants based on self-harm attempts. Also, the prevalence of self-harm between males and females has no significant difference (62.9 % vs. 50.0 %, *P* = 0.24). There was a significant difference between the age of those who reported self-harm and other (14.5 (2.3) vs. 12.6 (1.9), *P* = 0.001), and self-harm prevalence increased in higher age groups(*p* = 0.01).

**Table 2:** Comparison of characteristics’ participants based on self-harm attempt

Characteristic	Sub-characteristic	Self-harm		***P***-Value
		Yes	No	
		Number (%)	Number (%)	
**Gender**	**Male**	44(62.9)	26 (37.1)	0.24
	**Female**	14(50.0)	14(50.0)	
**Age**	**8-10**	2(40.0)	3 (60.0)	0.003
	**10-12**	11(35.5)	20(64.5)	
	**12-14**	18(62.1)	11(37.9)	
	**14-16**	11(73.3)	4(26.7)	
	**16-18**	16(88.9)	2(11.1)	
**Educational status**	**Student**	40(54.8)	33(45.2)	0.05
	**Dropped out of school**	18(72.0)	7(28)	
**Mental disorder**	**Have**	24(85.7)	4(14.3)	0.001
	**No have**	34(48.6)	36(51.4)	
**Parental life status**	**Both alive**	39(52.0)	36(48.0)	0.02
	**Only the mother is alive**	12(75.0)	4(25.0)	
	**Only the father is alive**	5(100)	0(0.0)	
	**Both died**	2(100)	0(0.0)	
**Parental divorced**	**Yes**	6(75.0)	2(25.0)	0.34
	**No**	52(57.8)	38(42.2)	
**Paternal education Level**	**Illiterate**	33(76.7)	10(23.3)	0.003
	**Literate**	21(43.8)	27(56.3)	
**Maternal education level**	**Illiterate**	28(73.7)	10(26.3)	0.02
	**Literate**	30(50.0)	30(50.0)	
**Communication with parents**	**Everyday**	50(57.5)	37(42.5)	0.33
	**Never or Low**	8(72.7)	3(27.3)	
**Drug abuse**	**Yes**	8(80.0)	2(20.0)	0.17
	**drug rehabilitated**	10(71.4)	4(28.6)	
	**Never**	40(54.1)	34(45.9)	
**History of suicide in the relatives**	**Yes**	15(83.3)	3(16.7)	0.02
	**No**	43(53.8)	37(46.3)	
**History of self-harm in the relatives**	**Yes**	21(77.8)	6(22.2)	0.02
	**No**	37(52.1)	34(47.9)	

The parental educational level in self-harmed children was significantly lower than other groups both for the paternal and maternal educational level (*P* = 0.003, 0.02 respectively). Children with mental disorders were significantly more likely to have self-harmful behaviors (*P* = 0.001). Concerning the life status of the participant’s parents, children who had more attempted self-harm were those whose parents had both died, and conversely, less self-harmed were those whose parents were both alive (*P* = 0.02). History of self-injury behaviors or suicide in relatives were the significant risk factors in self-harmed children (*P* = 0.02 for both). Finally, significant variables were enrolled in binary logistic regression analysis on detecting more important risk factors for self-harm.

Table 3 shows the results of logistic regression analysis. The results demonstrate that having a mental disorder (OR = 6.3, *P* = 0.002), losing parents (OR = 4.4, *P* = 0.01), history of suicide in relatives (OR = 4.3, *P* = 0.03), history of self-harm in relatives (OR = 3.2, *P* = 0.001), low educated parents (OR = 4.2, *P* = 002, OR = 2.8, *P* = 0.02 for father and mother respectively), and age-increasing (OR = 1.5, *P* = 0.001) were the most important risk factors.

**Table 3:** The results of the regression analysis for determining predicting factors of self-harm

Characteristic	B	OR	CI_95 %_ of OR	*P*-Value
Mental Disorder	1.84	6.35	2.00-19.11	0.002
Parental life status (losing parents)	1.47	4.38	1.36–14.12	0.01
Suicide in relatives (have)	1.45	4.31	1.15–16.02	0.03
Self-harm in relatives (have)	1.16	3.21	1.16–8.91	0.001
Father’s education (low educated)	1.44	4.24	1.71–10.52	0.002
Mother’s education (low educated)	1.03	2.81	1.15–6.76	0.02
Age	0.48	1.5	1.2–1.9	0.001

**Fig. 1:**
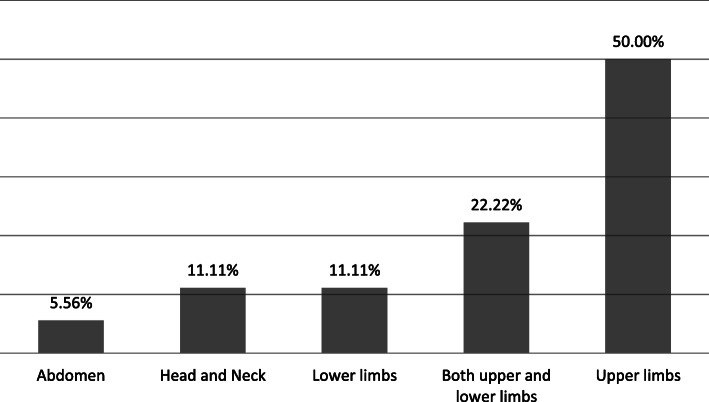
Distribution of self-harm by parts of the body in self-harmed street children

Table 4 shows the frequency of participants’ responses to the SHI questionnaire. As it demonstrates, the more frequently reported self-harm behaviors in physical injuries were hitting one’s self (26.5 %), self-starvation (23.5 %), and cutting one’s self (21.4 %). The most common part of self-injury was the upper limb (50 %), especially the forearms and wrists. After that, respectively, both upper and lower limbs, lower limbs, head and neck, and abdomen were more common (Fig. 1).

**Table 4:** Frequency of participants’ responses to the SHI questionnaire

	Questions: In the past 4 months, have you intentionally done any of following behaviors?	Answer	
		**Yes**	**No**
		**Number (%)**	**Number (%)**
1	Taking overdose(s)	7(7.10)	91(92.92)
2	Cutting one’s self	21(21.40)	77(78.60)
3	Burning one’s self	6(6.10)	92(93.90)
4	Hitting one’s self	26(26.50)	72(73.50)
5	Banging one’s head	16(16.30)	82(83.70)
6	Abusing alcohol	2(2.00)	96(98.00)
7	Driven recklessly	0(0.00)	98(100.00)
8	Superficially scratching self	16(16.30)	82(83.70)
9	Preventing wounds from healing	17(17.30)	81(82.70)
10	Making medical problems worse deliberately	11(11.20)	87(88.80)
11	Being promiscuous	1(1.00)	97(99.00)
12	Setting up a relationship for rejection	17(17.30)	81(82.70)
13	Abusing prescription medication	13(13.30)	85(86.70)
14	Distancing one’s self from God	29(29.60)	69(70.40)
15	Engaging in emotionally abusive relationships	16(16.30)	82(83.70)
16	Engaging in sexually abusive relationships	2(2.00)	96(98.00)
17	Losing a job deliberately	8(8.20)	90(91.80)
18	Attempting suicide	3(3.10)	95(96.90)
19	Exercising an injury deliberately	3(3.10)	95(96.90)
20	Engaging in self-defeating thoughts	19(19.40)	79(80.60)
21	Self-starvation	23(23.50)	75(76.50)
22	Abusing laxatives	1(1.00)	97(99.00)

In psychosocial injurious behaviors, the more frequent behaviors were God-distancing (29.6 %), engaging in self-defeating thoughts (19.4 %), setting up a relationship for rejection (17.3 %), and engaging in emotionally abusive relationships (16.3 %). There were no differences in the frequency of positive responses to each question regarding gender, except for sexual abuse that all of them were female.

Table 5 shows the distribution of frequency of positive response to the SHI questionnaire among participants who attempted self-harm. Based on the positive response to each question of the SHI questionnaire, only 8.6 % of participants had one self-harming behavior, and others expressed more than one. Assessment of frequency of different items of SHI questionnaire showed that in children who reported self-harm, the median of positive items was 3, and its interquartile range was 2–7.

**Table 5:** Distribution of frequency of positive response to SHI questionnaire in participants who attempted self-harm

Frequency of positive response	Number	Percent	Cumulative Percent
1	5	8.6	8.6
2	16	27.6	36.2
3	13	22.4	58.6
4 and more	24	41.4	100.0
Total	58	100.0	

## Discussion

The result of this survey showed that about 59 % of street children had attempted self-harm. There was no difference between girls and boys in terms of the frequency of self-harm. The age-increasing was directly associated with more self-harm. Losing the parents, a history of mental disorders, a history of self-harm or suicide in relatives, and lower education of parents (as a social determinant of health) had important factors for self-harm in children.

Some studies were conducted to determine the prevalence of self-harm in different age groups and various populations. In a similar study, a lower prevalence rate of self-harm was reported than the result of our study about street children’s self-harm. It should be noted, the participants in most studies were a little older than our range, and a few studies assessed self-harm in lower age children.

Mohammadpour et al. determined in their study, which was conducted in 2009, that the incidence of self-harm in Iranian teenagers educating at the second-grade high school (mean age of 16 years old) was about 4.8 % per year, and this phenomenon was associated with their age, cigarette and alcohol abuse [14]. Furthermore, Gholamzadeh et al. in 2017 in Fars Province (Iran) conducted a five-year population-based study on people with the mean age of 25 years old who had a history of self-harm. They demonstrated that self-harm was more common in lower education levels and males. Moreover, the most common parts for harming were the posterior side of the body, like shoulders, and these self-harming actions mostly are non-suicidal [15]. However, the difference between our and their study results may be due to differences in age of participants. In another study conducted by Fakhari A et al. in 2007 in Tabriz (Iran), it was determined that the most frequent self-harming among high school students is carving. Also, there was a significant relationship between self-harming and smoking, and alcohol use [16].

Research by Nada-raja et al. in 2004 among USA youth (aged 26 years) reported lifetime prevalence of self-harm was 13 %, and 9 % of them had at least one attempted suicide. They demonstrated that people who begin self-harm after a while are more likely to tend suicide [3]. A school-based study in Norway conducted to determine the changes in the prevalence of self-harm and its related factors in the adolescents, in 2002 and 2018, showed self-harm prevalence increased from 4 to 16 %, and it was higher for girls, higher among younger adolescents [17].

In a large population-based cohort study in Australia, the prevalence of self-injury in school pupils aged 14–15 years reported 8 %, and also girls (10 %) more than boys (6 %) reported self-harm [18].

Different surveys in the United States for detecting the prevalence of self-harm in life reported 20–37 % among people aged 14–16 years old and 7–8 % for children aged 11–13 years old [19].

According to the three-phase population-based cohort study conducted by Morgan et al. in 2017 among the British children between 10 and 19 years old, they observed a higher prevalence of self-harm in girls and even a sharp increase in the prevalence of self-harm among girls in 13–16 years old girls between 2011 and 2014 [20].

According to the relevant factors, early detection of pubertal and/or psychiatric disorders such as drug abuse either in children or their parents, identification of vulnerable children and families, support for abused or orphaned children by integrating the required care in the national primary health care program, are some of the policies and programs that can lead to the prevention of the harmful behaviors in the community.

Our results determined that the loss of parents is a risk factor for self-harm; this phenomenon could be due to the parenting style and the supervisory role of them in the families. Based on the Iranian social culture, in the absence of one parent, the other usually takes on the role of two parents, but in the absence of two parents, the risk is higher.

Familiarity with the correct patterns of parenting and resolving conflicts in the family by referring to the scientific support centers is one of the most important points in parenting. In addition, parental educational level was determined as an important factor in parenting and the occurrence of harmful behaviors in siblings.

The limitations of this study were the inability to access some street children for representation of the research sample. Still, this study was novel based on a few studies conducted about self-harm in street children and child labor situations.

## Conclusion

The result of this study indicated that more than half of street children had attempted self-harm, which shows that the prevalence of self-harm in these children is high and requires serious consideration. Children characteristics such as the mental disorders and parental factors such as parental education level, losing parents, and history of self-harm in relatives were important risk factors for incidence of self-harm in children.

## Data Availability

The data are not publicly available due to the privacy of research participants. The datasets used and/or analyzed during the current study are available from the corresponding author on reasonable request.

## Abbreviations

SHI: Self-harm Inventory questionnaire
UNICEF: The United Nations Children's Fund
WHO: World Health Organization
